# Supplementary material for: Clustering and halogen effects enabled red/near-infrared room temperature phosphorescence from aliphatic cyclic imides
Source: Nat Commun. 2022 May 12;13:2658. doi: 10.1038/s41467-022-30368-7 (PMC9098632; doi:10.1038/s41467-022-30368-7)
Supplement: Supplementary file 1 — Supplementary Information [file 41467_2022_30368_MOESM1_ESM.pdf]

Supplementary Information

Clustering and halogen effects enabled red/near-infrared room temperature phosphorescence from aliphatic cyclic imides

Tianwen Zhu[1], Tianjia Yang[1], Qiang Zhang[1] & Wang Zhang Yuan[1]*

[1]School of Chemistry and Chemical Engineering, Frontiers Science Center for Transformative Molecules, Shanghai Key Lab of Electrical Insulation and Thermal Aging, Shanghai Jiao Tong University, Shanghai 200240, China.
*E-mail: wzhyuan@sjtu.edu.cn

Supplementary Methods

Single crystal cultivation of SI and DBMI

SI and DBMI were commercially obtained and recrystallized twice before use. Single crystals of SI and DBMI were obtained by a solvent evaporation method from their methanol solutions. The collected crystals were dried under vacuum at 80 ºC overnight.

Characterization data of SI

^{1}H NMR (500 MHz, DMSO-d_6, δ, ppm) 11.05 (s, 1H), 2.57 (s, 4H). ^{13}C NMR (126 MHz, DMSO-d_6, δ, ppm) 179.85, 29.98. HRMS ($C_4H_5NO_2$): *m/z* 100.0396 (M+H$^+$, calcd 100.0399).

Characterization data of DBMI

^{1}H NMR (500 MHz, DMSO-d_6, δ, ppm) 11.49 (s, 1H). ^{13}C NMR (126 MHz, DMSO-d_6, δ, ppm) 165.77, 130.27. HRMS ($C_4HBr_2NO_2$): *m/z* 255.8432, 253.8448, 257.8417 (M+H$^+$, calcd 255.8433, 253.8453, 257.8412).

Synthesis of trans-2,3-dibromosuccinimide (DBSI)

DBSI was synthesized according to the procedures described in the literature[1]. MI (4.00 g, 40 mmol) was first dissolved in $CHCl_3$ (80 mL), then Br_2 (2.5 mL, 50 mmol) in $CHCl_3$ (20 mL) was added dropwise. The mixture was refluxed for 2.5 h, then allowed to cool down to room temperature over 1 h. Afterward, *n*-hexane was added into it, the yellow precipitates were collected after filtration and dried under vacuum at 40 ^{o}C overnight. Finally, such crude product was purified by column chromatography using methanol as the eluent to afford the white solids (5.61 g) in a yield of 55%. ^{1}H NMR (500 MHz, DMSO-d_6, δ, ppm) 12.21 (s, 1H), 5.40 (s, 2H). ^{13}C NMR (126 MHz, DMSO-d_6, δ, ppm) 170.74, 167.35, 133.76, 131.43. HRMS ($C_4H_3Br_2NO_2$): *m/z* 257.8586, 255.8603, 259.8571 (M+H$^+$, calcd 257.8589, 255.8610, 259.8569).

Synthesis of 1,3-diiodomaleimide (DIMI)

DIMI was synthesized according to the procedures described in the literature[2]. Firstly, DBMI (0.50 g, 2 mmol) was dissolved in AcOH (50 mL), and then NaI (0.88 g, 6 mmol) was added. The mixture was heated at 120 ^{o}C for 2 h, then allowed to cool down to room temperature. Consequently, the mixture was added into ice water, yellow precipitates were collected after filtration and dried under vacuum at 80 ^{o}C overnight. The crude product was further purified by column chromatography using DCM as the eluent to afford the orange-yellow solids (0.52 g) in a yield of 75%. ^{1}H NMR (500 MHz, DMSO-d_6, δ, ppm) 11.49 (s, 1H). ^{13}C NMR (126 MHz, DMSO-d_6, δ, ppm) 169.14, 120.52. HRMS ($C_4HI_2NO_2$): *m/z* 349.8172 (M+H$^+$, calcd 349.8176).

Single crystal cultivation of DBSI and DIMI

Single crystals of DBSI and DIMI were also obtained through a solvent evaporation process from their methanol solutions. The collected crystals were dried under vacuum at 80 ^{o}C overnight.

Preparation of PMMA doped films

PMMA powders (0.20 g) were added into DCM (3 mL) and stirred until being dissolved. Halogenated cyclic imides (DBSI, DBMI and DIMI) with varying weights (2.02, 10.52, 22.22 mg) were also dissolved in methanol (1 mL). The resulting solutions could be well mixed and then poured into the mold and placed in an oven at 45 ^{o}C for 12 h. The resulting films with varying doping fractions (1, 5, 10 wt%) were obtained for further characterization.

Synthesis of 1,3-bis(maleimide)propane (2MIP)

2MIP was synthesized according to the procedures described in the literature[3]. All of the following steps were conducted under N_2 atmosphere. Firstly, MA (12.26 g, 125 mmol) was dissolved in DMF (38 mL), and then DAP (5.3 mL, 63 mmol) in DMF (7 mL) was added dropwise. The mixture was stirred for 1 h at 40 ^{o}C. Next, acetic anhydride (14.2 mL, 150 mmol) was added, followed by sodium carbonate (2.50 g, 24 mmol). The mixture was further allowed to stir for 3 h at 55 ^{o}C, then allowed to cool down to room temperature, precipitated, washed with ice-cold water and purified using a SepaBean machine (Santai Technologies Inc., China) to afford the white solids (9.65 g) in a yield of 66%. ^{1}H NMR (500 MHz, DMSO-d_6, δ, ppm) 7.02 (s, 4H), 3.38 (t, J=7.3 Hz, 4H), 1.77 (p, J=7.3 Hz, 2H). ^{13}C NMR (126 MHz, DMSO-d_6, δ, ppm) 171.42, 135.01, 35.32, 27.27. HRMS ($C_{11}H_{10}N_2O_4$): *m/z* 235.0710 (M+H$^+$, calcd 235.0720).

Synthesis of 1,3-bis(bromomaleimide)propane (2BMIP)

2BMIP was synthesized via the bromination of 2MIP. Firstly, 2MIP (1.15 g, 10 mmol) was dissolved in DCM (20 mL), then Br_2 (1.2 mL, 22 mmol) was added dropwise under N_2 atmosphere. The mixture was refluxed for 3 h, then allowed to cool down to room temperature. Afterward, *n*-hexane was added into it, the yellow precipitates were collected after filtration, washed with saturated sodium bicarbonate solution

for several times and dried under vacuum at 80 °C overnight. The crude product was further purified by a SepaBean machine (Santai Technologies Inc., China) to afford the yellowish solids (1.88 g) in a yield of 48%. ^{1}H NMR (500 MHz, DMSO-d_6, δ, ppm) 7.45 (s, 2H), 3.44 (t, J=7.2 Hz, 4H), 1.82 (p, J=7.2 Hz, 2H). ^{13}C NMR (126 MHz, DMSO-d_6, δ, ppm) 169.33, 166.01, 133.06, 130.90, 36.37, 26.89. HRMS ($C_{11}H_8Br_2N_2O_4$): *m/z* 349.8172 (M+H$^+$, calcd 392.8909, 390.8930, 394.8889).

Single crystal cultivation of 2MIP and 2BMIP

Single crystals of 2MIP and 2BMIP were also obtained through a solvent evaporation process from their ethanol and DCM solutions, respectively. The collected crystals were dried under vacuum at 50 ºC overnight.

Synthesis of monothiosuccinimide (MTSI)

MTSI was synthesized according to the procedures described in the literature[4]. Firstly, SI (1.00 g, 10 mmol) was dissolved in toluene (20 mL), and then Lawesson's reagent (1.01 g, 2.5 mmol) was added. The mixture was refluxed for 12 h, then allowed to cool down to room temperature. The filtrate was collected and further purified by column chromatography using DCM as the eluent to afford the yellowish solids (0.21 g) in a yield of 18%. ^{1}H NMR (500 MHz, DMSO-d_6, δ, ppm) 12.64 (s, 1H), 3.03-2.97 (m, 2H), 2.69-2.63 (m, 2H). ^{13}C NMR (126 MHz, DMSO-d_6, δ, ppm) 216.12, 181.91, 40.99, 31.15. HRMS (C_4H_5NOS): *m/z* 116.0170 (M+H$^+$, calcd 116.0171).

Synthesis of dithiosuccinimide (DTSI)

DTSI was synthesized according to the procedures described in the literature[5]. Firstly, SI (0.49 g, 5 mmol) was dissolved in toluene (20 mL), and then Lawesson's reagent (2.02 g, 5 mmol) was added. The mixture was stirred for 3 h at 80 °C, then allowed to cool down to room temperature, precipitated, and further

purified by column chromatography using DCM/*n*-hexane (1/1) as the eluent to afford the yellow solids (0.23 g) in a yield of 35%. ^{1}H NMR (500 MHz, DMSO-d_6, δ, ppm) 13.81 (s, 1H), 3.12 (s, 4H). ^{13}C NMR (126 MHz, DMSO-d_6, δ, ppm) 217.59, 44.02. HRMS ($C_4H_5NS_2$): *m/z* 131.9950 (M+H$^+$, calcd 131.9942).

Single crystal cultivation of MTSI and DTSI

Single crystals of MTSI and DTSI were also obtained through a solvent evaporation process from their DCM and methanol solutions, respectively. The collected crystals were dried under vacuum at 50 ºC overnight.

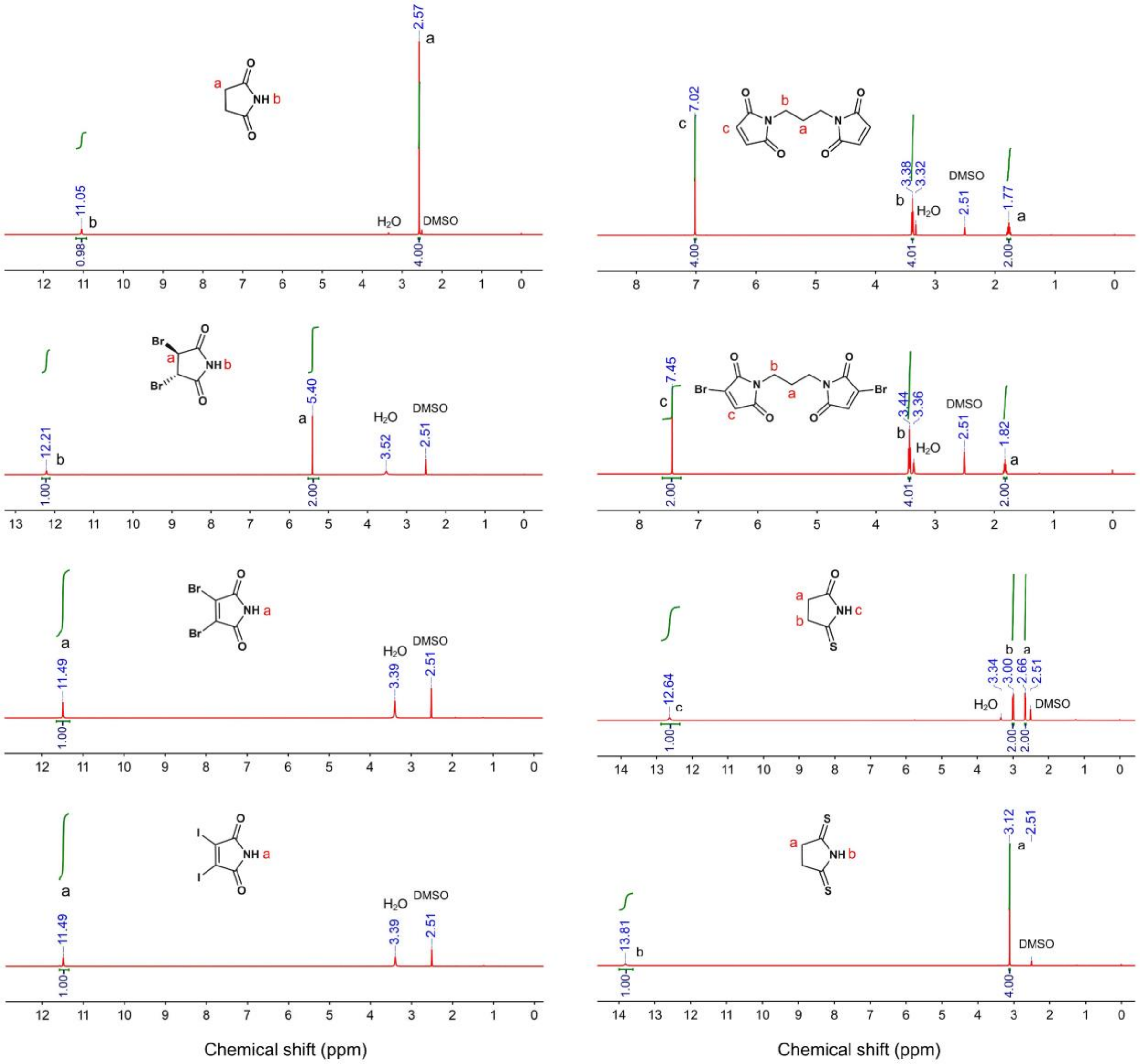

Supplementary Fig. 1 ^{1}H NMR characterization. ^{1}H NMR spectra of SI, DBSI, DBMI, DIMI, 2MIP, 2BMIP, MTSI and DTSI in DMSO-d_6.

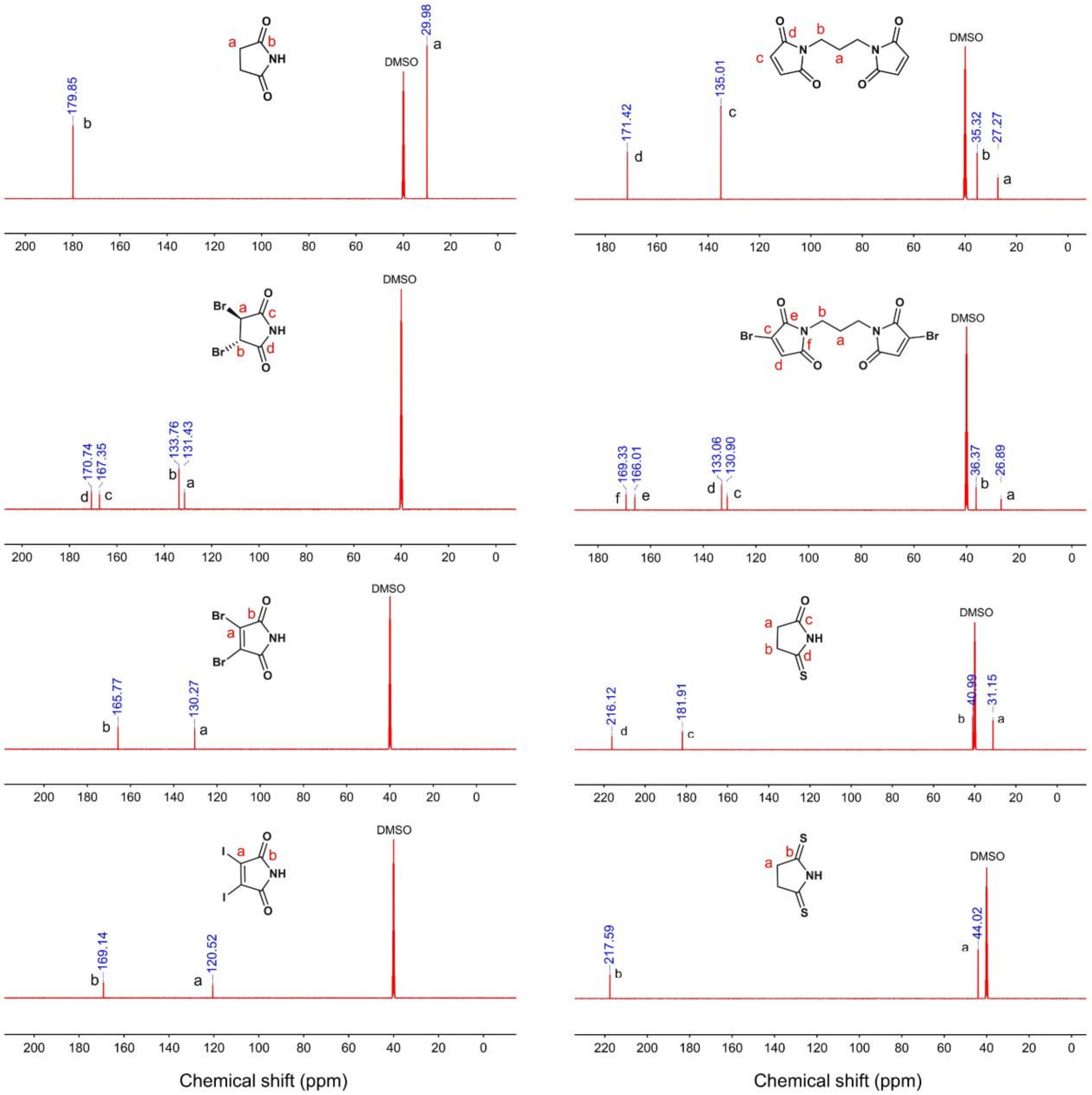

Supplementary Fig. 2 ^{13}C NMR characterization. ^{13}C NMR spectra of SI, DBSI, DBMI, DIMI, 2MIP, 2BMIP, MTSI, and DTSI in DMSO-d_6.

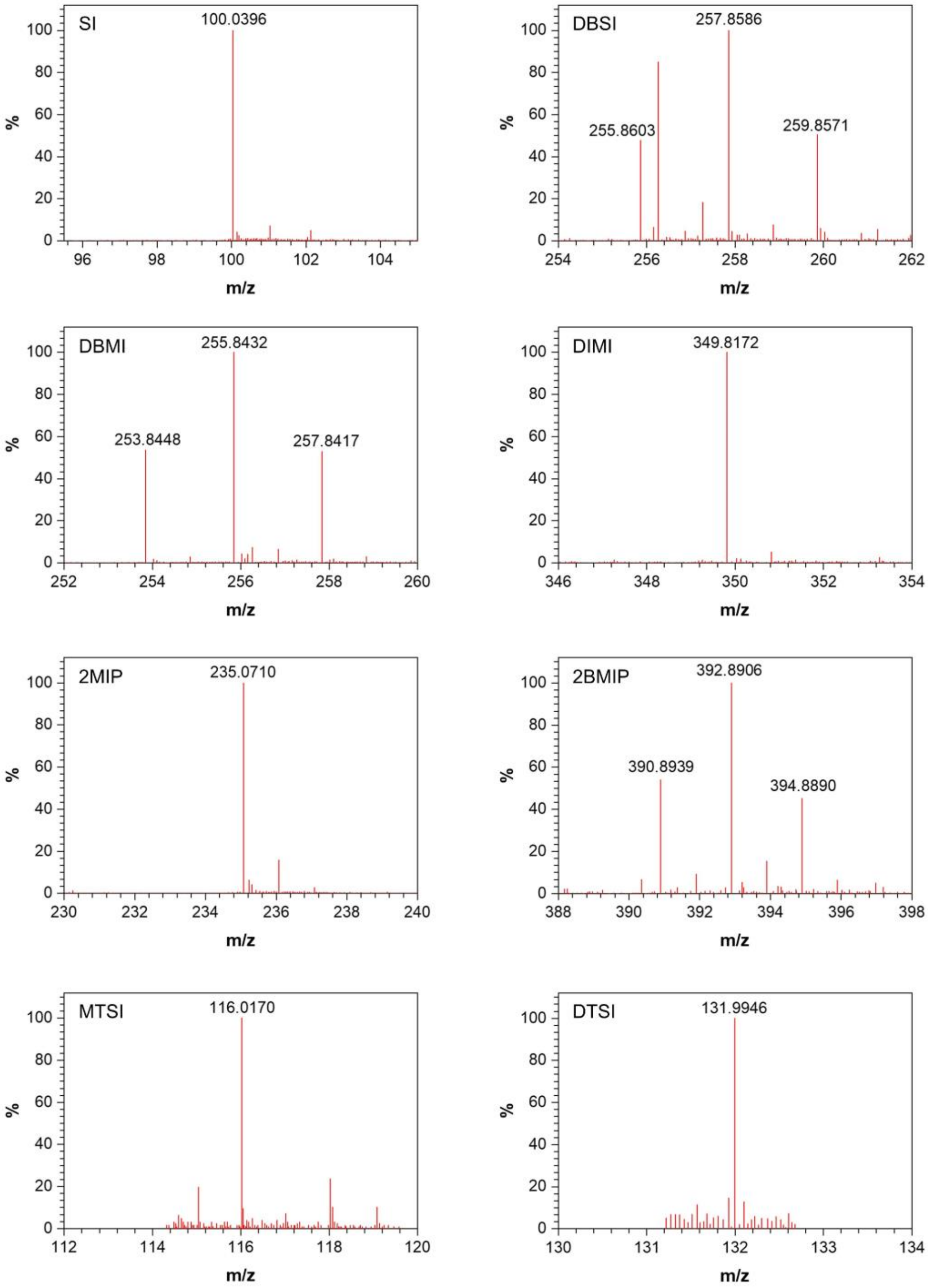

Supplementary Fig. 3 HRMS characterization. HRMS spectra of SI, DBSI, DBMI, DIMI, 2MIP, 2BMIP, MTSI, and DTSI.

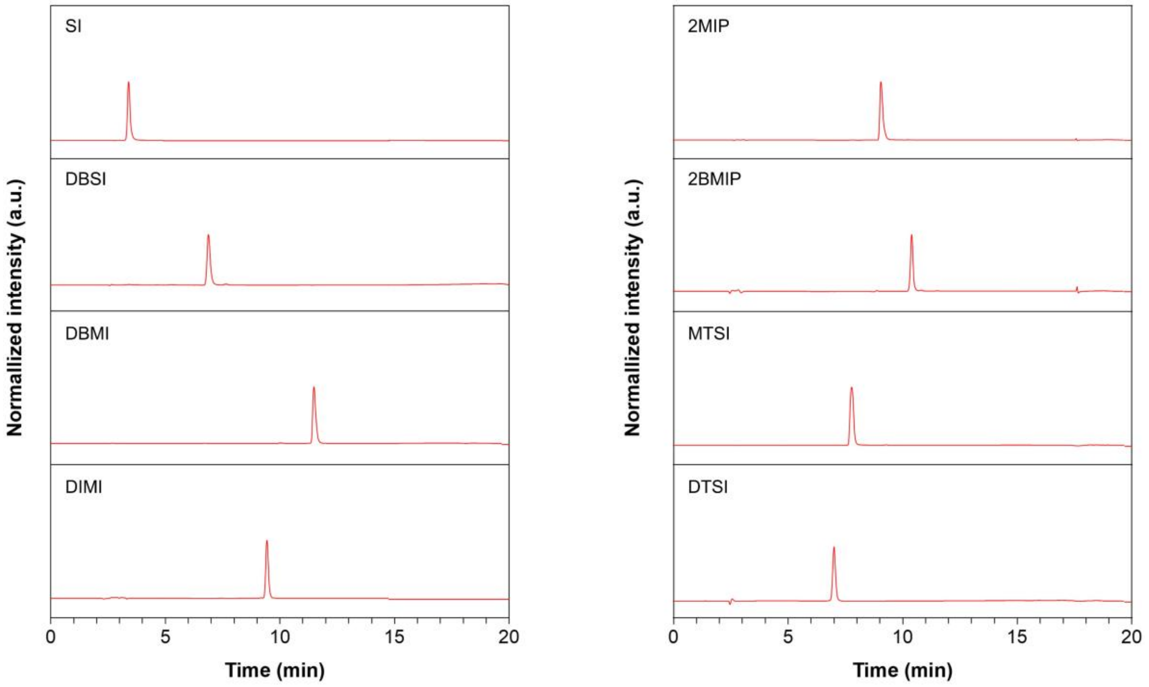

Supplementary Fig. 4 HPLC characterization. HPLC analysis spectra of SI, DBSI, DBMI, DIMI, 2MIP, 2BMIP, MTSI, and DTSI.

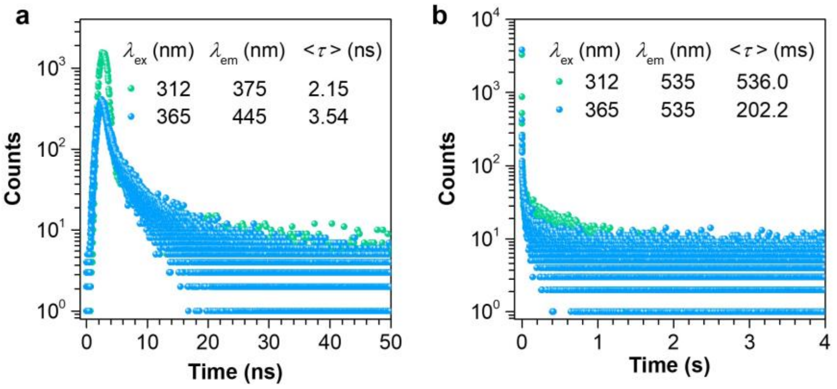

Supplementary Fig. 5 Lifetime profiles of SI. a Nanosecond and **b** microsecond scale lifetime profiles of SI single crystals.

Supplementary Table 1. ns- and ms-Scale lifetimes of SI single crystals.

λ_{ex} [nm]	λ_{em} [nm]	A_1 [%]	τ_1 [ns]	A_2 [%]	τ_2 [ns]	A_3 [%]	τ_3 [ns]	τ [ns]
312	375	68.92	0.41	31.08	6.02	-	-	2.15
365	445	44.75	0.97	55.25	5.63	-	-	3.54
λ_{ex} [nm]	λ_{em} [nm]	A_1 [%]	τ_1 [ms]	A_2 [%]	τ_2 [ms]	A_3 [%]	τ_3 [ms]	τ [ms]
312	535	9.31	1.79	9.72	37.43	80.98	657.2	536.0
365	535	13.92	2.27	32.91	36.12	53.17	357.3	202.2

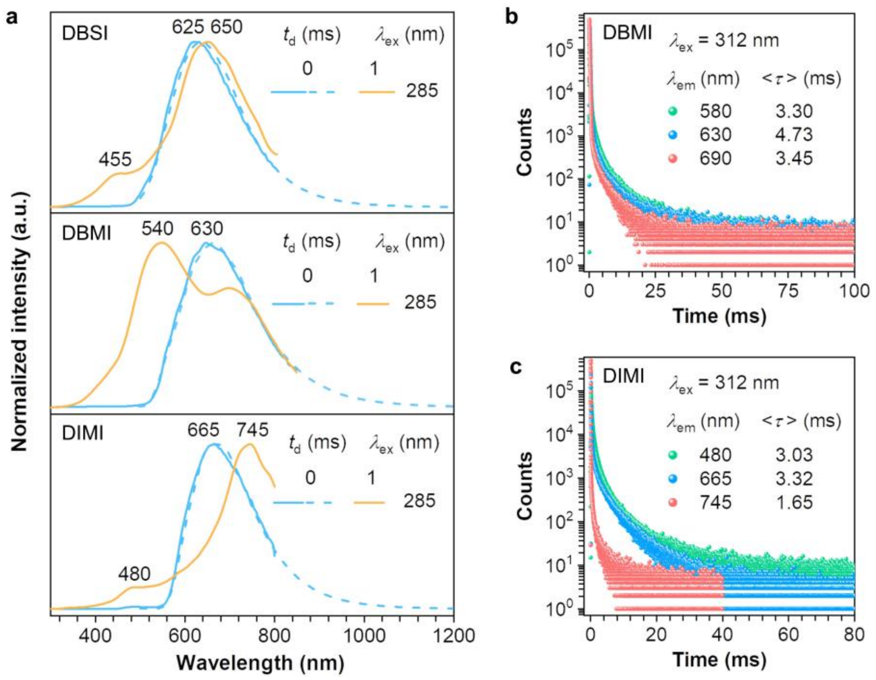

Supplementary Fig. 6 Photophysical properties of DBSI, DBMI, and DIMI. a Prompt (t_d = 0 ms) and delayed (t_d = 1 ms) emission spectra of DBSI, DBMI, and DIMI single crystals under 285 nm UV irradiation. Lifetime profiles of **b** DBMI and **c** DIMI single crystals under 312 nm UV irradiation.

Supplementary Table 2. Phosphorescence lifetimes of DBSI, DBMI, and DIMI single crystals.

λ_{ex} [nm]	λ_{em} [nm]	A_1 [%]	τ_1 [ms]	A_2 [%]	τ_2 [ms]	A_3 [%]	τ_3 [ms]	A_4 [%]	τ_4 [ms]	τ [ms]
					DBSI					
312	625	46.52	1.71	53.48	12.86	-	-	-	-	7.67
	650	0.60	0.10	28.21	1.77	45.75	7.33	25.44	25.50	10.34
365	625	0.78	0.10	24.95	1.65	42.21	7.74	32.05	31.21	13.68
	650	38.06	0.05	31.56	3.13	30.38	30.14	-	-	10.16
					DBMI					
312	580	20.15	0.35	55.73	1.87	24.11	9.09	-	-	3.30
	630	2.53	0.10	33.86	0.93	44.84	3.59	18.77	14.92	4.73
	690	14.15	0.17	43.88	1.70	41.97	6.39	-	-	3.45
365	595	0.48	0.003	56.64	0.15	22.29	2.05	20.58	19.69	4.59
	630	20.24	0.17	30.94	1.89	48.82	26.17	-	-	13.40
	685	19.92	0.18	45.99	1.98	34.09	16.08	-	-	6.43
					DIMI					
312	480	34.18	0.62	50.02	2.44	15.79	10.10	-	-	3.03
	665	20.58	0.42	45.72	2.18	33.70	6.65	-	-	3.32
	745	7.69	0.05	53.94	0.50	38.38	3.57	-	-	1.65
365	485	24.17	0.52	47.38	2.70	28.45	16.24	-	-	6.03
	665	25.15	0.83	74.85	2.13	-	-	-	-	1.80
	740	3.52	0.05	54.05	0.57	42.43	4.60	-	-	2.26

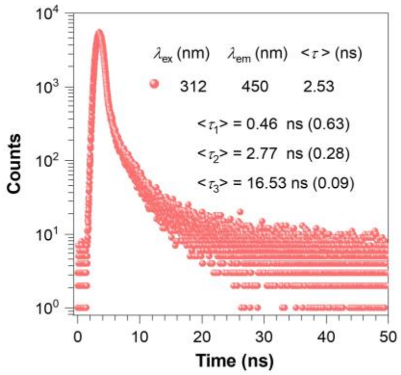

Supplementary Fig. 7 Lifetime characterization. Nanosecond scale lifetimes of DBSI single crystals.

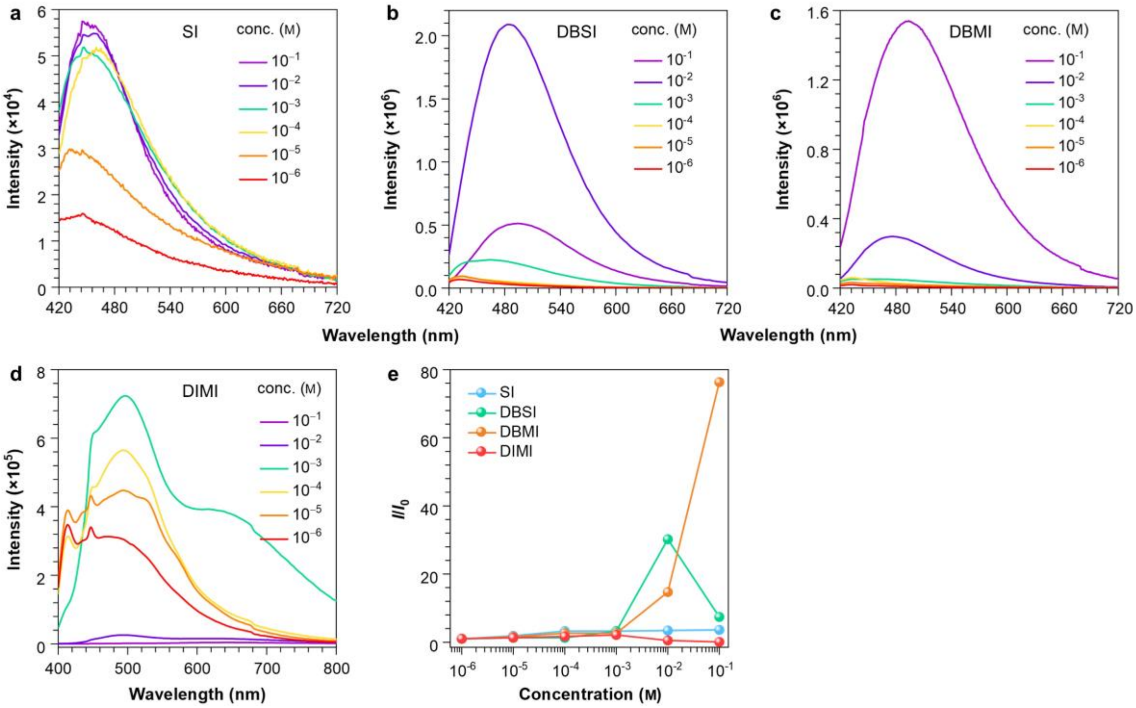

Supplementary Fig. 8 Emission spectra of SI, DBSI, DBMI, and DIMI/THF solutions. Emission spectra of **a** SI, **b** DBSI, **c** DBMI and **d** DIMI in THF at various concentrations (λ_{ex} = 365 nm). **e** Relative PL maximal intensities (I/I_0) of the THF solutions for the compounds at various concentrations.

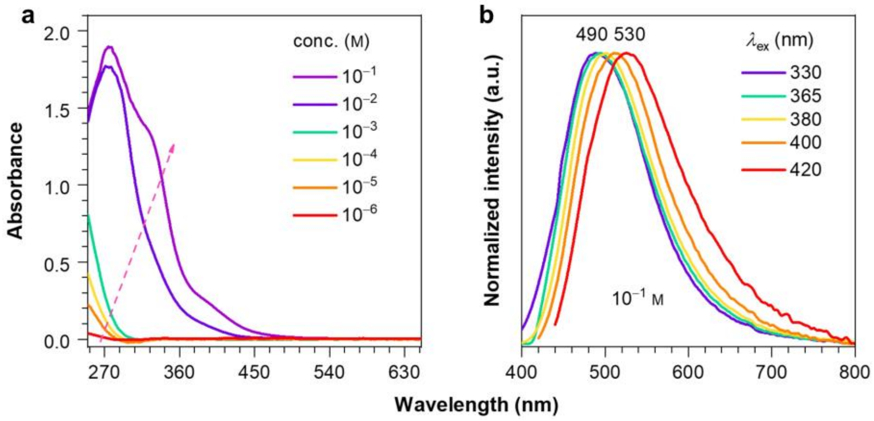

Supplementary Fig. 9 Photophysical properties of DBSI/THF solutions. a Absorption spectra of DBSI/THF solutions at various concentrations. **b** Normalized PL spectra of 10^{-1} M DBSI/THF solution with different λ_{ex}s.

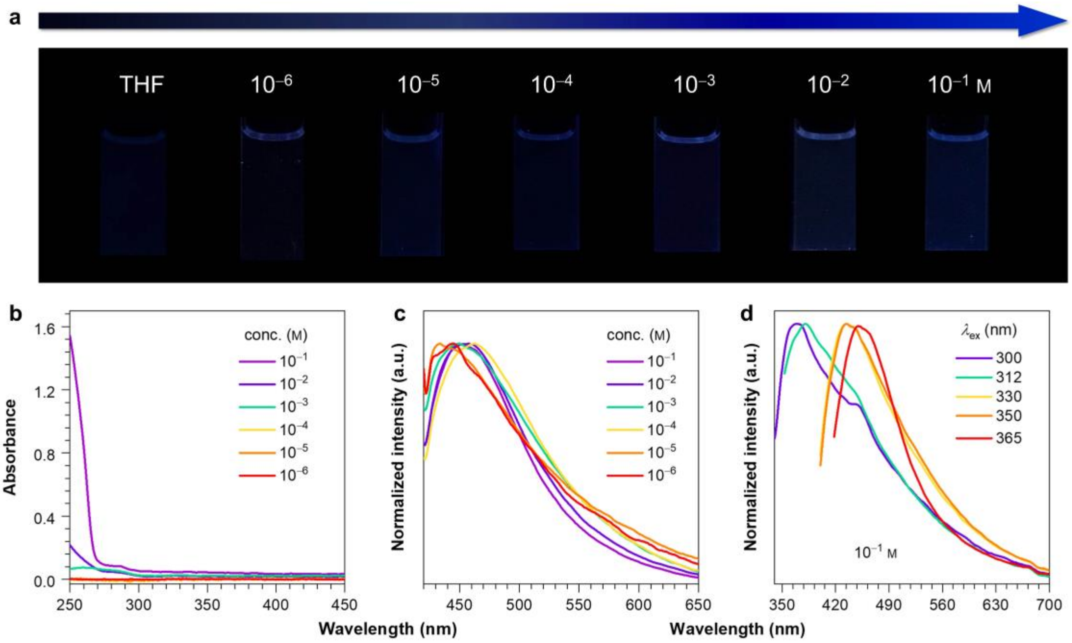

Supplementary Fig. 10 Photophysical properties of SI/THF solutions. a Photographs taken under 365 nm UV light, **b** absorption and **c** emission spectra (λ_{ex} = 365 nm) of different of SI/THF solutions. **d** Normalized PL spectra of 10^{-1} M SI/THF solution with different λ_{ex}s.

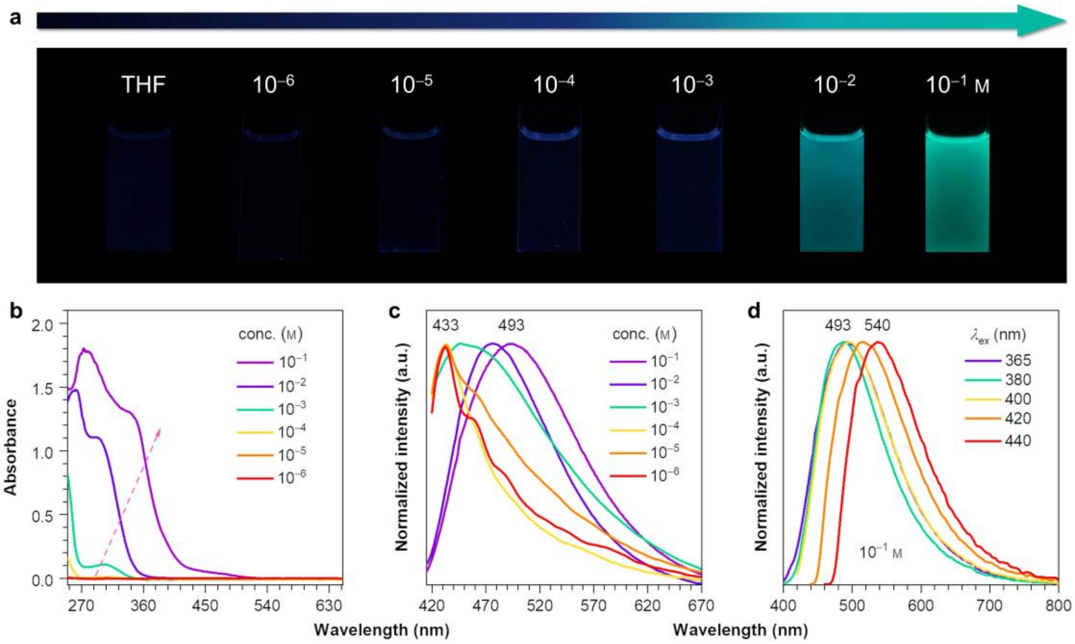

Supplementary Fig. 11 Photophysical properties of DBMI/THF solutions. a Photographs taken under 365 nm UV light, **b** absorption and **c** emission spectra (λ_{ex} = 365 nm) of different of DBMI/THF solutions. **d** Normalized PL spectra of 10^{-1} M DBMI/THF solution with different λ_{ex}s.

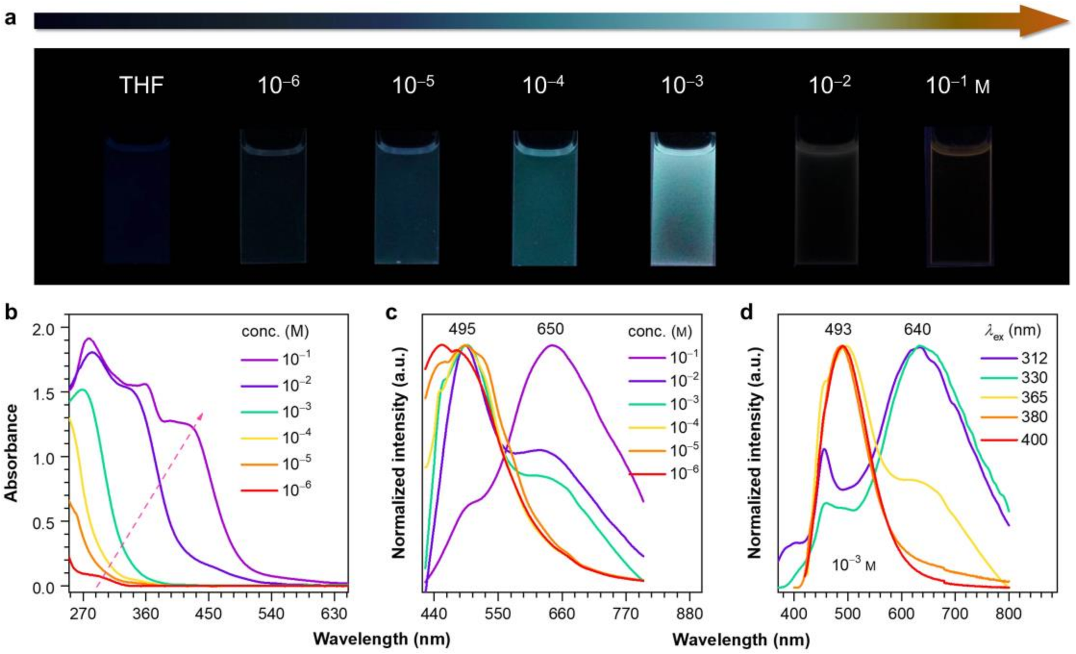

Supplementary Fig. 12 Photophysical properties of DIMI/THF solutions. a Photographs taken under 365 nm UV light, **b** absorption and **c** emission spectra (λ_{ex} = 365 nm) of different of DIMI/THF solutions. **d** Normalized PL spectra of 10^{-3} M DIMI/THF solution with different λ_{ex}s.

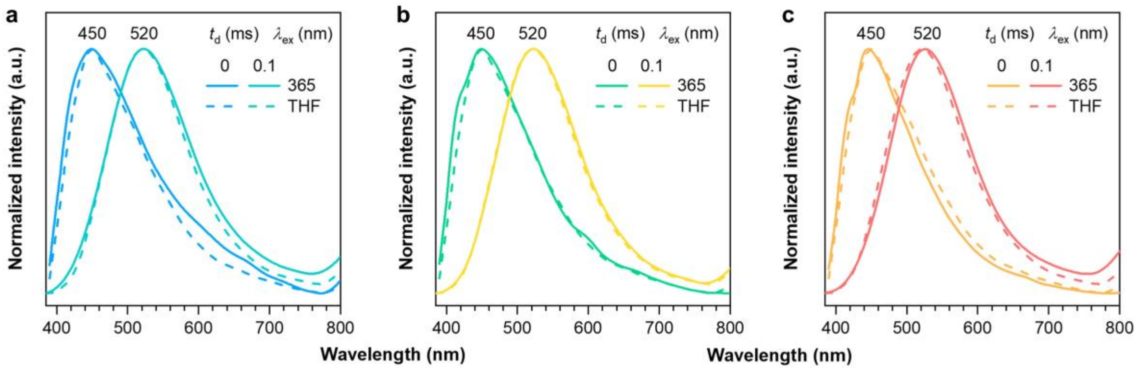

Supplementary Fig. 13 Emission spectra of dilute THF solutions of DBSI, DBMI, and DIMI at 77 K. Normalized prompt (t_d = 0 ms) and delayed (t_d = 0.1 ms) emission spectra (λ_{ex} = 365 nm) of 10^{-6} M THF solutions of **a** DBSI, **b** DBMI and **c** DIMI at 77 K.

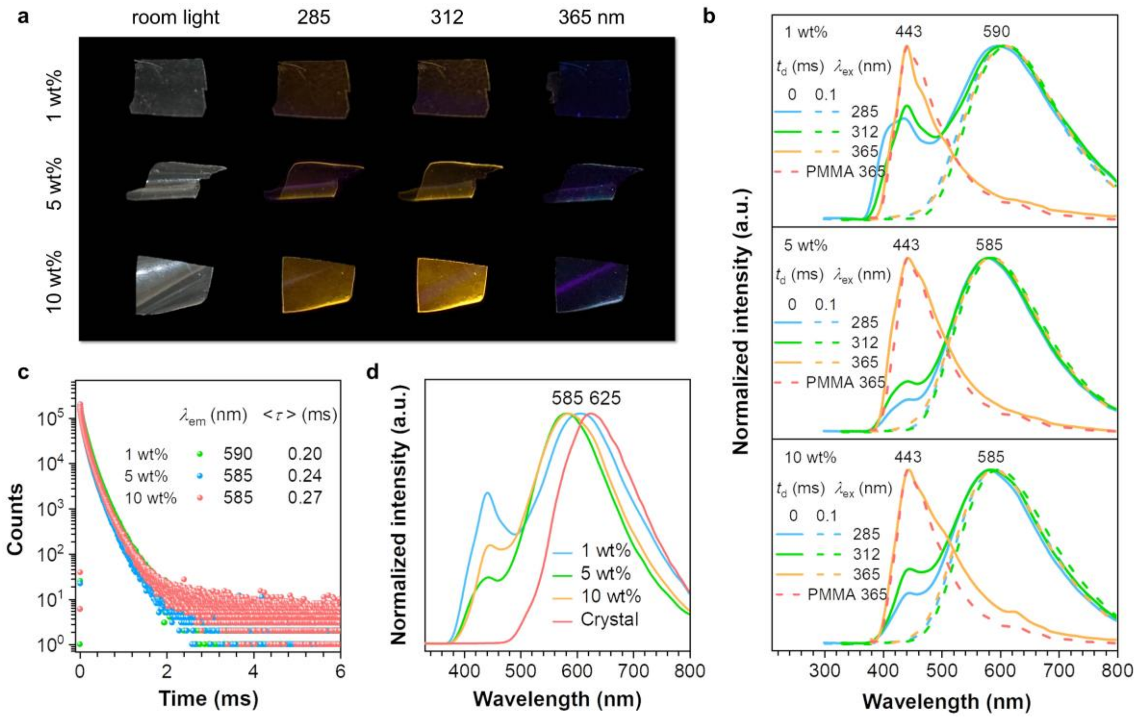

Supplementary Fig. 14 Photophysical properties of DBSI/PMMA films. a Photographs taken under room light or varying UV lights and **b** prompt (t_d = 0 ms) and delayed (t_d = 0.1 ms) emission spectra with varying λ_{ex}s of DBSI/PMMA films with different doping fractions. **c** Lifetime profiles of DBSI/PMMA films with different doping fractions (λ_{ex} = 312 nm). **d** Prompt emission spectra of DBSI crystals and DBSI/PMMA films with different doping fractions (λ_{ex} = 312 nm).

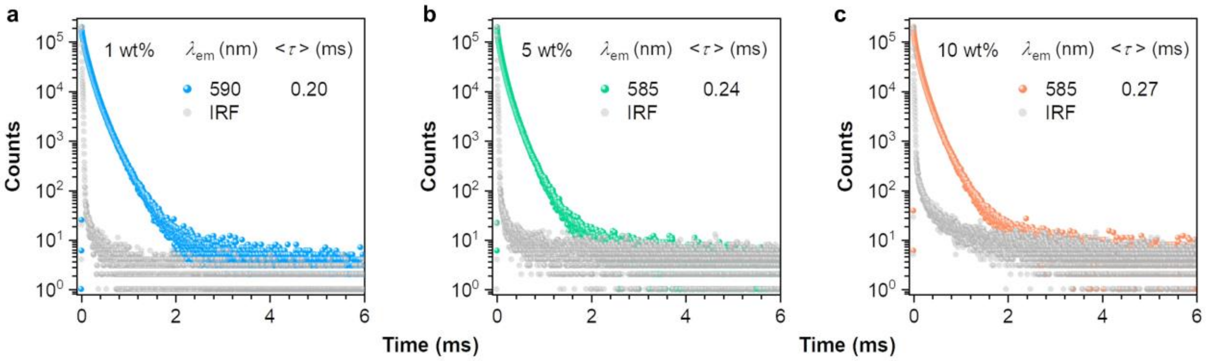

Supplementary Fig. 15 Lifetime profiles of DBSI/PMMA films. Lifetime profiles of **a** 1 wt%, **b** 5 wt%, and **c** 10 wt% DBSI/PMMA films and the IRF signal (λ_{ex} = 312 nm).

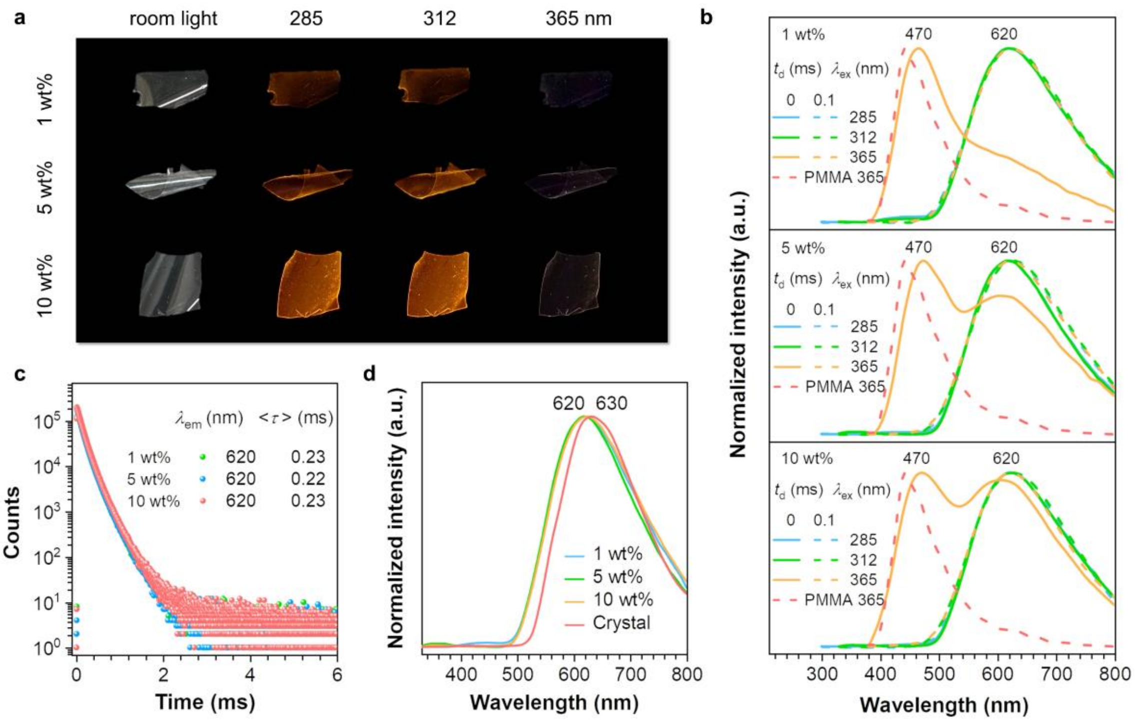

Supplementary Fig. 16 Photophysical properties of DBMI/PMMA films. a Photographs taken under room light or varying UV lights and **b** prompt (t_d = 0 ms) and delayed (t_d = 0.1 ms) emission spectra with varying λ_{ex}s of DBMI/PMMA films with different doping fractions. **c** Lifetime profiles of DBMI/PMMA films with different doping fractions (λ_{ex} = 312 nm). **d** Prompt emission spectra of DBMI crystals and DBMI/PMMA films with different doping fractions (λ_{ex} = 312 nm).

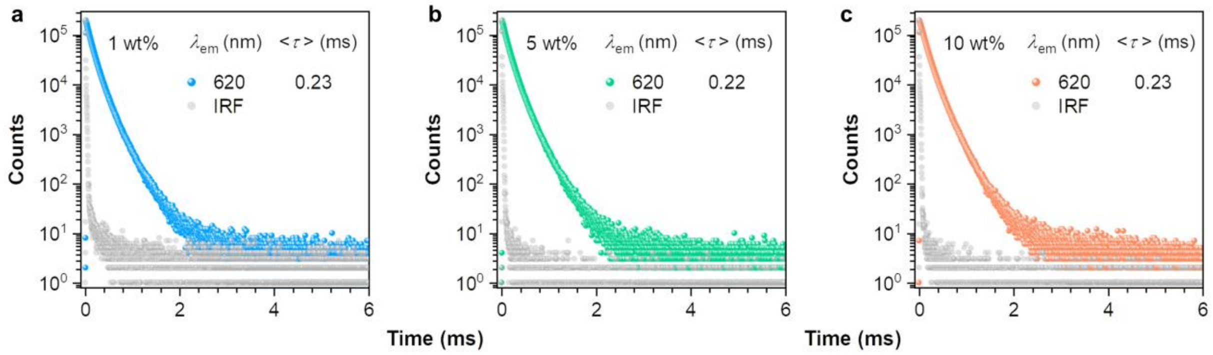

Supplementary Fig. 17 Lifetime profiles of DBMI/PMMA films. Lifetime profiles of **a** 1 wt%, **b** 5 wt%, and **c** 10 wt% DBMI/PMMA films and the IRF signal (λ_{ex} = 312 nm).

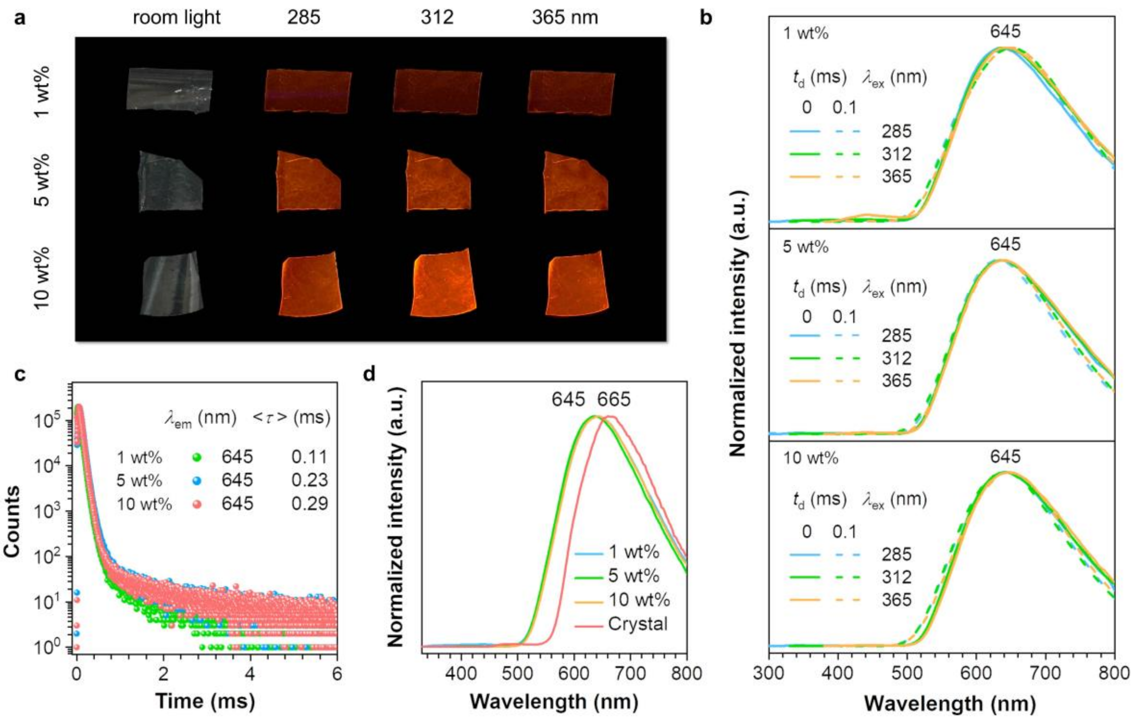

Supplementary Fig. 18 Photophysical properties of DIMI/PMMA films. a Photographs taken under room light or varying UV lights and **b** prompt (t_d = 0 ms) and delayed (t_d = 0.1 ms) emission spectra with varying λ_{ex}s of DIMI/PMMA films with different doping fractions. **c** Lifetime profiles of DIMI/PMMA films with different doping fractions (λ_{ex} = 312 nm). **d** Prompt (t_d = 0 ms) emission spectra of DIMI crystals and DIMI/PMMA films with different doping fractions (λ_{ex} = 312 nm).

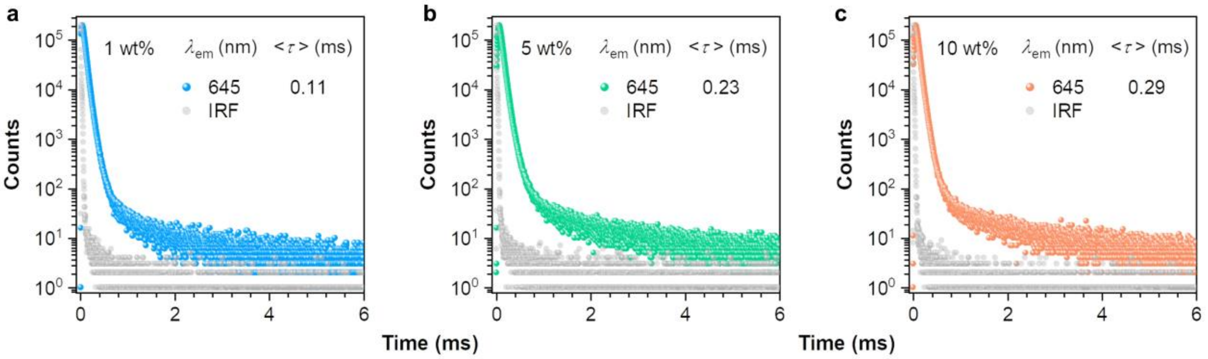

Supplementary Fig. 19 Lifetime profiles of DIMI/PMMA films. Lifetime profiles of **a** 1 wt%, **b** 5 wt%, and **c** 10 wt% DIMI/PMMA films and the IRF signal (λ_{ex} = 312 nm).

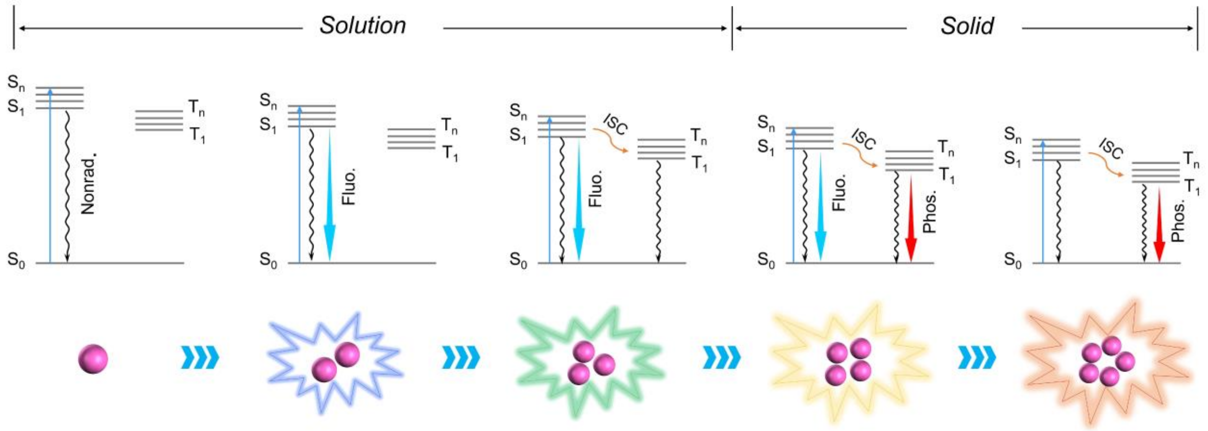

Supplementary Fig. 20 Luminescent mechanism illustration. Demonstration of the luminescent mechanism from solution to solid (ISC=intersystem crossing, Fluo. = fluorescence, Phos. = phosphorescence, Nonrad. = nonradiative transitions).

Supplementary Table 3. Single crystal data of SI, DBSI, DBMI, and DIMI at room temperature.

	SI	DBSI	DBMI	DIMI
CCDC	2063201	2063202	2063199	2063200
Formula	$C_4H_5NO_2$	$C_4H_3Br_2NO_2$	$C_4HBr_2NO_2$	$C_4HI_2NO_2$
Formula Weight	99.09	256.89	254.88	348.86
Meas. Temp. (K)	297 (2)	295 (2)	295 (2)	297 (2)
Wavelength (Å)	1.54178	1.54178	1.54178	1.54178
Space Group	Pbca	P21/c	P21/c	P21/c
Cell Length (Å)	a = 7.5350 (3)<br>b = 9.6228 (4)<br>c = 12.8889 (5)	a = 11.0960 (8)<br>b = 15.7858 (11)<br>c = 7.6768 (5)	a = 10.2448 (9)<br>b = 8.1117 (7)<br>c = 7.8144 (7)	a = 10.571 (2)<br>b = 8.3378 (18)<br>c = 8.2770 (19)
Cell Angle (°)	α = 90<br>β = 90<br>γ = 90	α = 90<br>β = 98.029 (2)<br>γ = 90	α = 90<br>β = 99.992 (4)<br>γ = 90	α = 90<br>β = 100.437 (10)<br>γ = 90
Cell Volume ($Å^3$)	934.55 (6)	1331.48 (16)	639.55 (10)	717.5 (3)
Z	8	8	4	4
Density (g cm^{-3})	1.409	2.563	2.647	3.230
F (000)	416.0	960.0	472.0	616.0
h_{max}, k_{max}, l_{max}	9, 11, 15	13, 19, 9	12, 9, 9	12, 10, 9
T_{min}, T_{max}	0.681, 0.753	0.128, 0.051	0.464, 0.753	0.264, 0.753

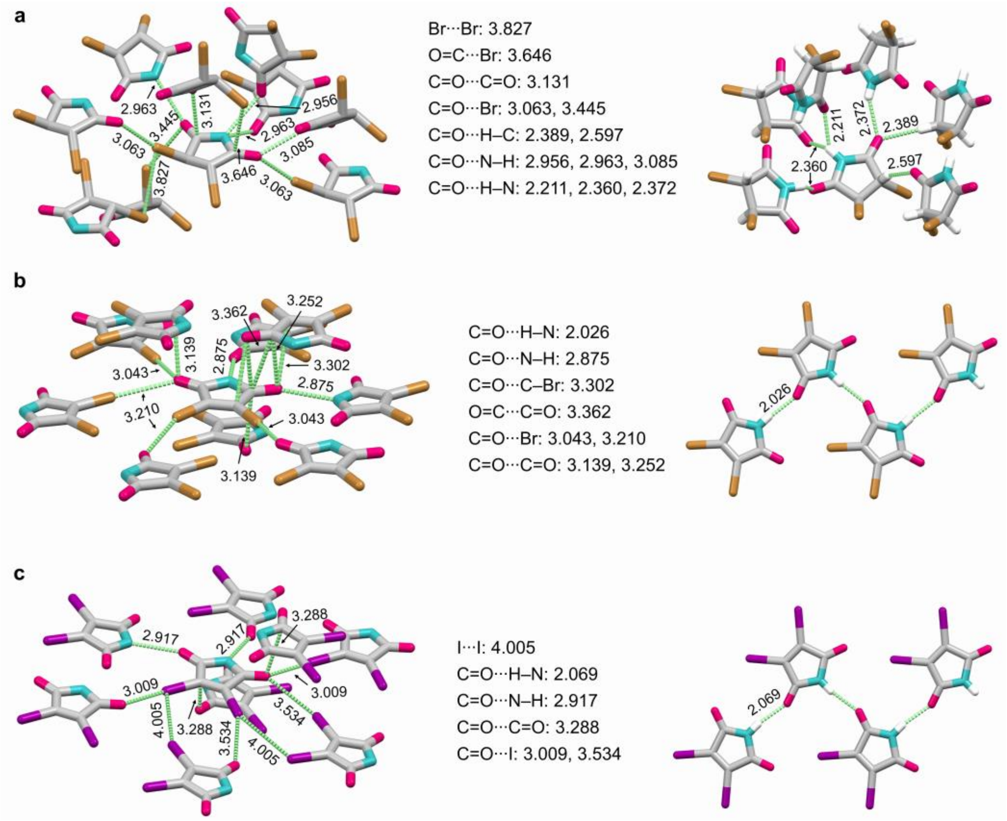

Supplementary Fig. 21 Single crystal structure analysis of DBSI, DBMI, and DIMI. Single crystal structure and fragmental molecular packing with denoted intermolecular interactions of **a** DBSI, **b** DBMI, and **c** DIMI.

Supplementary Table 4. Transition configurations of SI.[a]

Aggregation state	Excited state	Excitation energy [eV]	Transition configuration (%)
Monomer	S_1	4.9286	H→ L (69.025); H-1→ L+1 (14.298)
	T_1	4.4261	H→ L (67.671); H-1→ L+1 (19.265)
	T_2	4.9741	H-1→ L (60.116); H→ L+1 (36.340)
	T_3	5.0989	H-2→ L (67.708); H-3→ L+1 (16.519)
Dimer	S_1	4.9656	H-1→ L (47.165); H→ L+1 (50.138)
	T_1	4.4733	H-2→ L+2 (12.416); H-1→ L (46.266); H-1→ L+2 (10.380); H→ L+1 (48.763)
	T_2	4.4743	H-2→ L+3 (11.966); H-1→ L+1 (46.696); H→ L (48.307); H→ L+2 (11.241)
	T_3	5.0828	H-4→ L+1 (44.102); H-3→ L (42.727); H-2→ L (26.114); H-1→ L+2 (11.434); H→ L+3 (11.027)

[a] The matched triplet excited states that contain the same orbital transition components of S_1 are highlighted in red.

Supplementary Table 5. Transition configurations of DBSI.[a]

Aggregation state	Excited state	Excitation energy [eV]	Transition configuration (%)
Monomer	S_1	4.5220	H-4→ L (10.126); H-1→ L+1 (11.075); H→ L (64.594)
	T_1	4.0446	H-4→ L (11.468); H-1→ L+1 (14.615); H→ L (61.647)
	T_2	4.3594	H-3→ L (13.332); H-1→ L (39.457); H→ L+1 (47.014); H→ L+3 (11.006)
	T_3	4.6435	H-6→ L (17.817); H-2→ L (42.285)
Dimer	S_1	4.6863	H-2→ L (30.450); H-1→ L (54.068)
	T_1	4.2233	H-2→ L (29.339); H-2→ L+2 (10.321); H-1→ L (50.307); H-1→ L+1 (10.614); H-1→ L+2 (11.191)
	T_2	4.2435	H→ L+1 (56.020); H→ L+3 (13.717)
	T_3	4.5819	H-8→ L (14.551); H-3→ L (33.217); H-2→ L+2 (24.085); H-1→ L+1 (15.602); H-1→ L+2 (31.235)
	T_4	4.6098	H-5→ L+1 (15.503); H-2→ L+1 (28.438); H-1→ L+2 (14.564); H→ L+3 (40.422)
	T_5	4.8312	H-10→ L+2 (40.025); H-10→ L+6 (11.273); H-4→ L+2 (36.566); H-1→ L+4 (25.680)

[a] The matched triplet excited states that contain the same orbital transition components of S_1 are highlighted in red.

Supplementary Table 6. Transition configurations of DBMI.[a]

Aggregation state	Excited state	Excitation energy [eV]	Transition configuration (%)
Monomer	S_1	3.5281	H-1→ L (70.078)
	T_1	2.9588	H→ L (67.424)
	T_2	3.0891	H-1→ L (69.341)
	T_3	3.5886	H-4→ L (16.073); H→ L (68.272)
	T_4	3.8550	H-3→ L (57.907); H-1→ L+3 (12.798)
Dimer	S_1	3.6383	H-3→ L (11.723); H-2→ L+1 (68.427)
	T_1	2.8148	H-1→ L (68.208)
	T_2	2.8260	H→ L+1 (68.078)
	T_3	3.2072	H-2→ L+1 (69.027)
	T_4	3.2279	H-3→ L (68.290)
	T_5	3.7178	H-10→ L (16.586); H-9→ L (41.663); H-8→ L (10.518); H→ L (50.929)

[a] The matched triplet excited states that contain the same orbital transition components of S_1 are highlighted in red.

Supplementary Table 7. Transition configurations of DIMI.[a]

Aggregation state	Excited state	Excitation energy [eV]	Transition configuration (%)
Monomer	S_1	3.5070	H-2→ L (43.538); H-1→ L (52.056); H→ L (18.222);
	T_1	2.6466	H-7→ L (17.476); H→ L (67.866);
	T_2	3.1292	H-2→ L (58.952); H-1→ L (36.700);
	T_3	3.6700	H-5→ L (57.515); H-1→ L (32.224);
Dimer	S_1	3.2009	H→ L (70.369);
	T_1	2.5795	H-15→ L (16.917); H-2→ L (67.675);
	T_2	2.6278	H-14→ L+1 (17.221); H→ L+1 (67.702);
	T_3	3.1411	H-8→ L (44.731); H-7→ L (30.416); H-4→ L (24.903);
	T_4	3.1462	H-8→ L (16.125); H-7→ L (10.908); H-4→ L (10.701); H-4→ L+1 (25.525); H-3→ L+1 (51.516); H-1→ L+1 (30.902);
	T_5	3.1946	H-8→ L (11.937); H→ L (67.547);
	T_6	3.5541	H-5→ L (12.828); H-1→ L (67.279);

[a] The matched triplet excited states that contain the same orbital transition components of S_1 are highlighted in red.

Supplementary Table 8. ns- and ms-Scale lifetimes of 2MIP single crystals.

λ_{ex} [nm]	λ_{em} [nm]	A_1 [%]	τ_1 [ns]	A_2 [%]	τ_2 [ns]	A_3 [%]	τ_3 [ns]	A_4 [%]	τ_4 [ns]	τ [ns]
312	401	48.94	0.56	51.06	8.14	-	-	-	-	4.43
365	450	55.93	0.79	44.07	4.28	-	-	-	-	2.33
λ_{ex} [nm]	λ_{em} [nm]	A_1 [%]	τ_1 [ms]	A_2 [%]	τ_2 [ms]	A_3 [%]	τ_3 [ms]	A_4 [%]	τ_4 [ms]	τ [ms]
312	535	24.20	0.07	40.82	0.34	25.72	1.58	9.26	7.82	1.29
	695	53.91	0.02	18.00	0.08	11.82	0.80	16.27	4.50	0.85
365	545	18.86	0.10	44.36	1.15	36.78	10.94	-	-	4.55

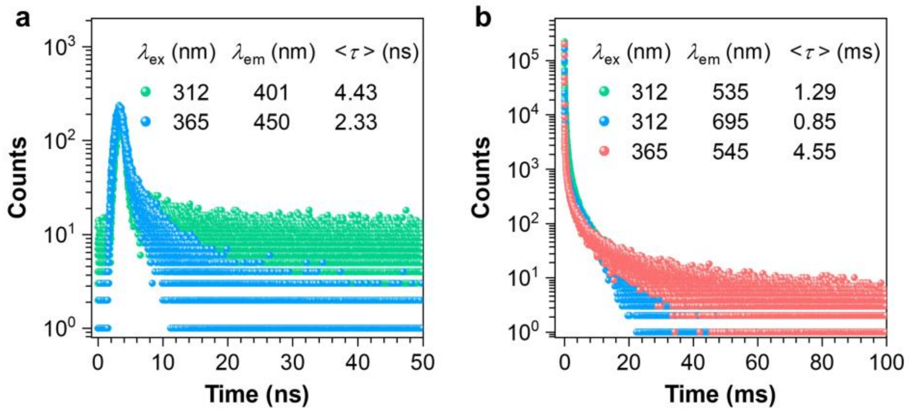

Supplementary Fig. 22 Lifetime profiles of 2MIP. a Nanosecond and **b** microsecond scale lifetime profiles of 2MIP single crystals.

Supplementary Table 9. Phosphorescence lifetimes of 2BMIP single crystals.

λ_{ex} [nm]	λ_{em} [nm]	A_1 [%]	τ_1 [ms]	A_2 [%]	τ_2 [ms]	A_3 [%]	τ_3 [ms]	τ [ms]
312	612	36.19	1.00	63.81	10.00	-	-	6.74
	675	35.33	1.08	64.67	9.91	-	-	6.79
365	612	1.96	0.10	39.11	1.62	58.93	12.64	8.08
	675	1.51	0.10	36.53	1.73	61.97	13.57	9.04

Supplementary Table 10. Dynamic photophysical parameters of the single crystals of 2MIP and 2BMIP.[a]

Sample	λ_f [nm]	λ_{p1} [nm]	λ_{p2} [nm]	Φ_c [%]	Φ_f [%]	Φ_p [%]	Φ_{p1} [%]	Φ_{p2} [%]	Φ_{isc} [%]	$<\tau>_f$ [ns]	$<\tau>_{p1}$ [ms]	$<\tau>_{p2}$ [ms]	k_{isc} [s^{-1}]	k_r^{p1} [s^{-1}]	k_r^{p2} [s^{-1}]	k_{nr}^{p1} [s^{-1}]	k_{nr}^{p2} [s^{-1}]
2MIP	401	535	695	0.7	0.2	0.5	0.3	0.2	71.4	4.43	1.29	0.85	3.2×10^5	2.3	2.4	772.9	1174.1
2BMIP	440	612	675	7.0	0	7.0	3.3	3.7	~100	-[b]	6.74	6.79	-	4.9	5.4	143.5	141.8

[a] λ_{ex} = 312 nm; $\Phi_c = \Phi_f + \Phi_p$; $\Phi_p = \Phi_{p1} + \Phi_{p2}$; $\Phi_{isc} = \Phi_p/(\Phi_p + \Phi_f)$; $k_{isc} = \Phi_p\Phi_f/(\Phi_p + \Phi_f)<\tau>_f$; $k_r^p = \Phi_p/<\tau>_p$; $k_{nr}^p = (1 - \Phi_p)/<\tau>_p$. λ_f and λ_p are the emission maxima of fluorescence and phosphorescence of the crystals. Φ_c, Φ_f, and Φ_p are the quantum efficiencies of total emission, fluorescence, and phosphorescence of the crystals, respectively.
[b] Cannot be traced.

Supplementary Table 11. Single crystal data of 2MIP and 2BMIP at room temperature.

	2MIP	2BMIP
CCDC	2094589	2125703
Formula	$C_{11}H_{10}N_2O_4$	$C_{11}H_8Br_2N_2O_4$
Formula Weight	234.21	392.01
Meas. Temp. (K)	296 (2)	296 (2)
Wavelength (Å)	1.54178	1.54178
Space Group	C2/c	C2/c
Cell Length (Å)	a = 19.3586 (13)<br>b = 6.7014 (5)<br>c = 9.1216 (6)	a = 16.511 (2)<br>b = 8.1581 (9)<br>c = 12.159 (3)
Cell Angle (°)	α = 90<br>β = 116.924 (3)<br>γ = 90	α = 90<br>β = 129.042 (5)<br>γ = 90
Cell Volume (Å^3)	1055.08 (13)	1272.1 (3)
Z	4	4
Density (g cm^{-3})	1.474	2.047
F (000)	488.0	760.0
h_{max}, k_{max}, l_{max}	23, 8, 10	19, 9, 14
T_{min}, T_{max}	0.642, 0.753	0.572, 0.753

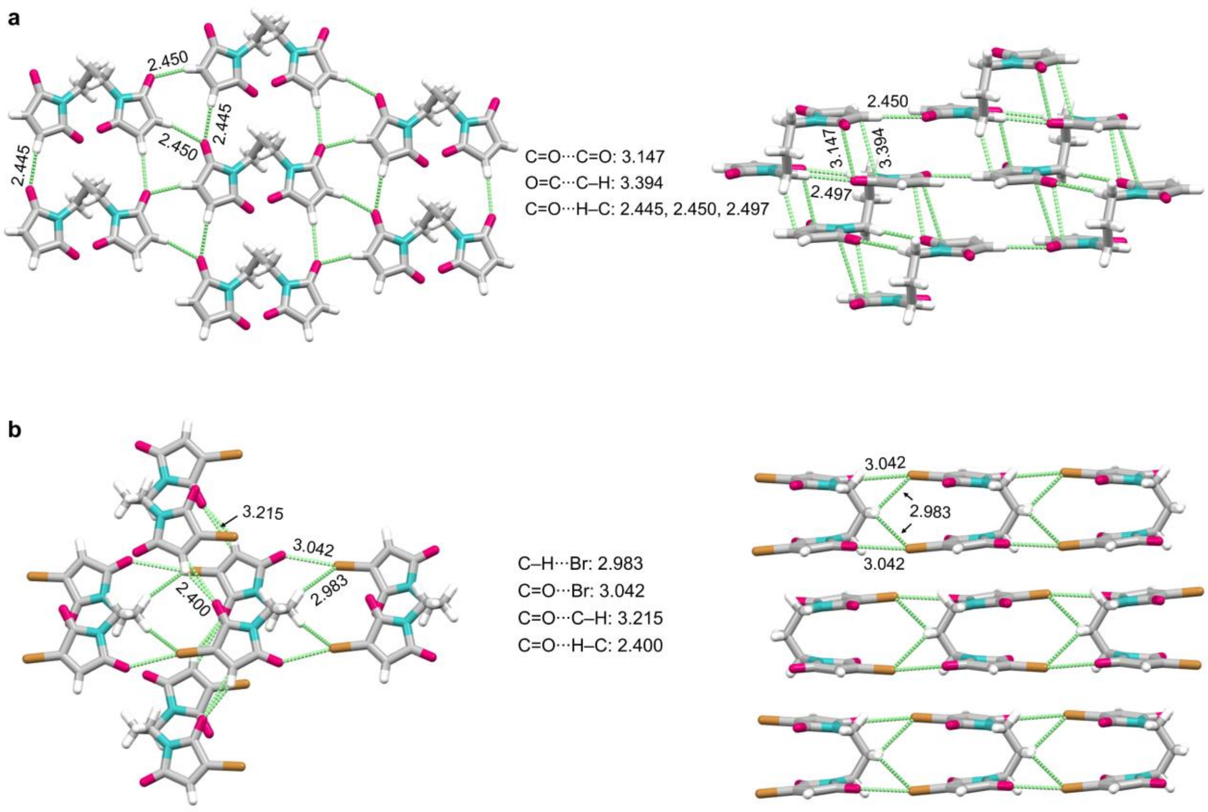

Supplementary Fig. 23 Single crystal structure analysis of 2MIP and 2BMIP. Single crystal structure and fragmental molecular packing with denoted intermolecular interactions of **a** 2MIP and **b** 2BMIP.

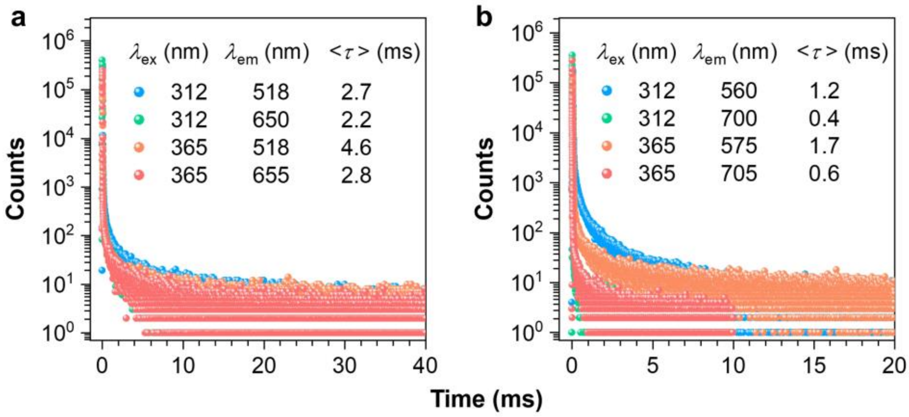

Supplementary Fig. 24 Lifetime characterization. Lifetime profiles of **a** MTSI and **b** DTSI single crystals.

Supplementary Table 12. Phosphorescence lifetimes of MTSI and DTSI single crystals.

λ_{ex} [nm]	λ_{em} [nm]	A_1 [%]	τ_1 [ms]	A_2 [%]	τ_2 [ms]	A_3 [%]	τ_3 [ms]	A_4 [%]	τ_4 [ms]	τ [ms]
					MTSI					
312	518	20.45	0.10	38.47	0.80	41.07	5.81	-	-	2.71
	650	14.32	0.10	39.10	0.76	46.58	4.01	-	-	2.18
365	518	10.87	0.10	35.52	0.93	53.61	8.03	-	-	4.65
	655	11.88	0.10	39.43	0.84	48.69	5.08	-	-	2.81
					DTSI					
312	560	0.41	0.01	27.46	0.13	41.94	0.60	30.19	3.11	1.23
	700	55.80	0.007	17.07	0.02	10.82	0.22	16.31	2.11	0.37
365	575	13.39	0.02	34.11	0.29	52.50	3.03	-	-	1.69
	705	15.63	0.02	43.65	0.21	40.71	1.32	-	-	0.63

Supplementary Table 13. Dynamic photophysical parameters of the single crystals of MTSI and DTSI.[a]

Sample	λ_{p1} [nm]	λ_{p2} [nm]	Φ_c [%]	Φ_f [%]	Φ_p [%]	Φ_{p1} [%]	Φ_{p2} [%]	Φ_{isc} [%]	$<\tau>_f$ [ns]	$<\tau>_{p1}$ [ms]	$<\tau>_{p2}$ [ms]	k_{isc} [s^{-1}]	k_r^{p1} [s^{-1}]	k_r^{p2} [s^{-1}]	k_{nr}^{p1} [s^{-1}]	k_{nr}^{p2} [s^{-1}]
MTSI	518	650	2.48	0	2.48	1.09	1.39	~100	-[b]	2.71	2.18	-	4.02	6.38	365.0	452.3
DTSI	560	700	0.43	0	0.43	0.17	0.26	~100	-[b]	1.23	0.37	-	1.38	7.03	811.6	2695.7

[a] λ_{ex} = 312 nm; $\Phi_c = \Phi_f + \Phi_p$; $\Phi_p = \Phi_{p1} + \Phi_{p2}$; $\Phi_{isc} = \Phi_p/(\Phi_p + \Phi_f)$; $k_{isc} = \Phi_p\Phi_f/(\Phi_p + \Phi_f) <\tau>_f$; $k_r^p = \Phi_p/<\tau>_p$; $k_{nr}^p = (1 - \Phi_p)/<\tau>_p$. λ_f and λ_p are the emission maxima of fluorescence and phosphorescence of the crystals. Φ_c, Φ_f, and Φ_p are the quantum efficiencies of total emission, fluorescence, and phosphorescence of the crystals, respectively.

[b] Cannot be traced.

Supplementary Table 14. Single crystal data of MTSI and DTSI at room temperature.

	MTSI	DTSI
CCDC	2125705	2125708
Formula	C_4H_5NOS	$C_4H_5NS_2$
Formula Weight	115.15	131.21
Meas. Temp. (K)	298 (2)	299 (2)
Wavelength (Å)	1.54178	1.54178
Space Group	P21/c	C2/m
Cell Length (Å)	a = 9.9619 (18)<br>b = 7.2447 (12)<br>c = 7.6031 (13)	a = 11.8851 (17)<br>b = 6.9058 (15)<br>c = 7.7927 (16)
Cell Angle (°)	α = 90<br>β = 97.818 (12)<br>γ = 90	α = 90<br>β = 110.292 (12)<br>γ = 90
Cell Volume (Å^3)	543.62 (16)	599.9 (2)
Z	4	4
Density (g cm^{-3})	1.407	1.453
F (000)	240.0	272.0
h_{max}, k_{max}, l_{max}	11, 8, 9	14, 8, 9
T_{min}, T_{max}	0.540, 0.753	0.540, 0.753

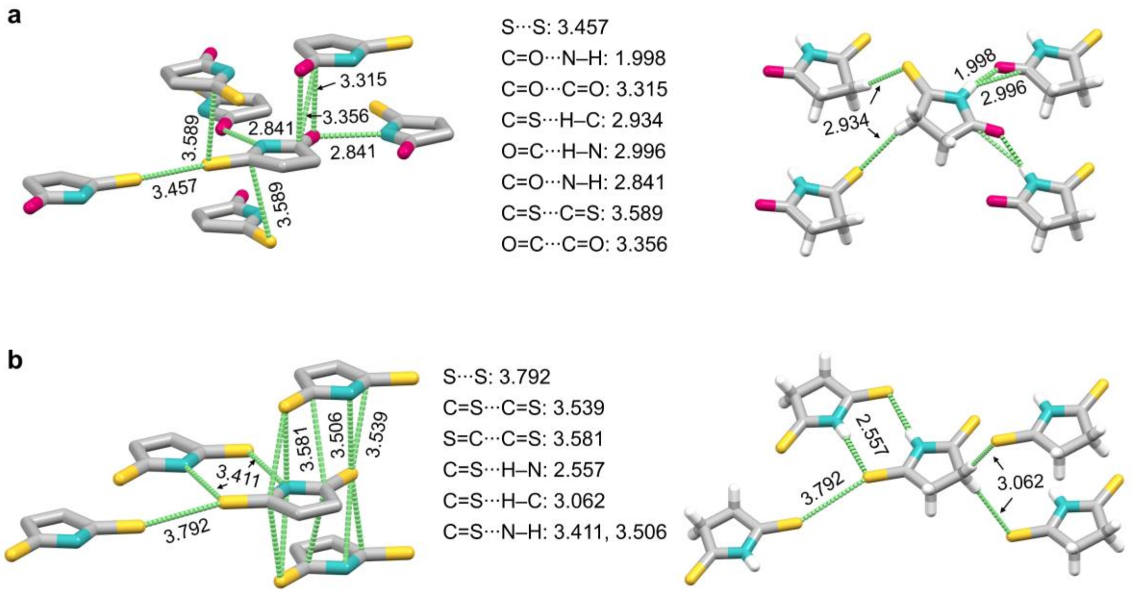

Supplementary Fig. 25 Single crystal structure analysis of MTSI and DTSI. Single crystal structure and fragmental molecular packing with denoted intermolecular interactions of **a** MTSI and **b** DTSI.

Supplementary References

1. Marculescu, C. et al. Aryloxymaleimides for cysteine modification, disulfide bridging and the dual functionalization of disulfide bonds. *Chem. Commun*. **50**, 7139–7142 (2014).

2. Forte, N. et al. Tuning the hydrolytic stability of next generation maleimide cross-linkers enables access to albumin-antibody fragment conjugates and tri-scFvs. *Bioconjugate Chem*. **29**, 486–492 (2018).

3. Lorenzini, R. G. & Sotzing, G. A. Furan/imide Diels–Alder polymers as dielectric materials. *J. Appl. Polym. Sci*. **131**, 40179 (2014).

4. Bishop, J. E., Dagam, S. A. & Rapoport, H. Synthesis and characterization of monothiosuccinimides. *J. Org. Chem*. **54**, 1876–1883 (1989).

5. Yde, B. et al. Studies on organophosphorus compounds XLVII preparation of thiated synthons of amides, lactams and imides by use of some new p,s-containing reagents. *Tetrahedron* **40**, 2047–2052 (1984).
